# Evaluation of Biomonitoring Data from the CDC National Exposure Report in a Risk Assessment Context: Perspectives across Chemicals

**DOI:** 10.1289/ehp.1205740

**Published:** 2012-12-11

**Authors:** Lesa L. Aylward, Christopher R. Kirman, Rita Schoeny, Christopher J. Portier, Sean M. Hays

**Affiliations:** 1 Summit Toxicology LLP, Falls Church, Virginia, USA; 2 Summit Toxicology LLP, Orange Village, Ohio, USA; 3 Office of Research and Development, U.S. Environmental Protection Agency, Washington, DC, USA; 4 National Center for Environmental Health/Agency for Toxic Substances and Disease Registry, Atlanta, Georgia, USA; 5 Summit Toxicology LLP, Lyons, Colorado, USA

**Keywords:** biomonitoring, Biomonitoring Equivalents, blood, cancer risk, CDC National Exposure Report, hazard quotient, NHANES, risk assessment, urine

## Abstract

Background: Biomonitoring data reported in the *National Report on Human Exposure to Environmental Chemicals* [NER; Centers for Disease Control and Prevention (2012)] provide information on the presence and concentrations of > 400 chemicals in human blood and urine. Biomonitoring Equivalents (BEs) and other risk assessment–based values now allow interpretation of these biomonitoring data in a public health risk context.

Objectives: We compared the measured biomarker concentrations in the NER with BEs and similar risk assessment values to provide an across-chemical risk assessment perspective on the measured levels for approximately 130 analytes in the NER.

Methods: We identified available risk assessment–based biomarker screening values, including BEs and Human Biomonitoring-I (HBM-I) values from the German Human Biomonitoring Commission. Geometric mean and 95th percentile population biomarker concentrations from the NER were compared to the available screening values to generate chemical-specific hazard quotients (HQs) or cancer risk estimates.

Conclusions: Most analytes in the NER show HQ values of < 1; however, some (including acrylamide, dioxin-like chemicals, benzene, xylene, several metals, di-2(ethylhexyl)phthalate, and some legacy organochlorine pesticides) approach or exceed HQ values of 1 or cancer risks of > 1 × 10^–4^ at the geometric mean or 95th percentile, suggesting exposure levels may exceed published human health benchmarks. This analysis provides for the first time a means for examining population biomonitoring data for multiple environmental chemicals in the context of the risk assessments for those chemicals. The results of these comparisons can be used to focus more detailed chemical-specific examination of the data and inform priorities for chemical risk management and research.

Large population-representative biomonitoring studies such as the *National Report on Human Exposure to Environmental Chemicals* [Centers for Disease Control and Prevention (CDC) 2012]—hereafter referred to as the National Exposure Report (NER)—and other national biomonitoring efforts, such as those conducted in Canada and in Germany, are providing valuable data on the prevalence and concentrations of chemicals in biological matrices such as blood or urine from individuals in the general population. These measured concentrations provide an integrated reflection of exposures that may occur via multiple routes and pathways. For this and other reasons, biomonitoring is increasingly being relied upon as a state-of-the-art tool for exposure assessment for environmental chemicals (Sexton et al. 2004). The NER provides unparalleled data on several hundred analytes in a representative sample of the U.S. general population. These data are a potentially rich source of information for risk managers and researchers looking to identify and study chemical exposures in the general population.

Biomonitoring studies can establish exposure levels across a study population and provide a means to compare exposures across population groups by age, sex, ethnicity, or other demographic descriptors. Biomonitoring results can also be used to establish research priorities, to measure trends in exposure over time and to verify the efficacy of selected pollution controls and other public health policy actions. There are limitations in biomonitoring data in that they are generally single time point measures. Moreover, as noted by the CDC (2005),

[T]he presence of a chemical does not imply disease. The levels or concentrations of the chemical are more important determinants of the relation to disease, when established in appropriate research studies, than the detection or presence of a chemical.

The significance of the measured concentrations of chemicals in the context of existing toxicology data and risk assessments can be assessed if chemical-specific, biomonitoring-based risk assessment values are available. Such risk assessment values would ideally be based on robust data sets relating adverse effects to biomarker concentrations in human populations (e.g., the historical use of a “blood lead level of concern” by the CDC and other organizations). However, development of such epidemiologically based values is a resource- and time-intensive effort, and in practice, data to support such assessments exist for only a few chemicals. As an interim approach, the concept of Biomonitoring Equivalents (BEs) has been developed, and guidelines for the derivation and communication of these values have been published (Hays et al. 2007, 2008a; LaKind et al. 2008).

A BE is defined as the concentration or range of concentrations of a chemical or its metabolites in a biological matrix (blood, urine, or other matrix) that is consistent with an existing noncancer health–based exposure guidance value such as a reference dose (RfD) or tolerable or acceptable daily intake (TDI or ADI) or with a cancer-based exposure guidance value such as a risk-specific dose (e.g., the dose associated with a 1 × 10^–4^ cancer risk) (Hays et al. 2008a). BEs are intended to be used as screening tools to provide an assessment of which chemical biomarkers are present at levels below, near, or above concentrations consistent with existing risk assessments and exposure guidance values. BEs allow for the translation of conventional risk assessment guidance to the evaluation of exposure information provided by biomonitoring data. Comparison of biomarker concentrations to corresponding BE values can be used to guide the evaluation of multiple exposures in a population and to set priorities for research or reduction in exposures.

BE values have now been derived for approximately 90 compounds included in the NER analyte list (Table 1; Angerer et al. 2011). Public health risk–based values in terms of biomarker concentrations for a number of additional analytes are available from several other sources [including the German Human Biomonitoring Commission (2012); reviewed by Angerer et al. (2011)].

**Table 1:** Risk assessment exposure guidance values (with year of derivation), corresponding screening BEs, and NER GMs and 95th percentiles for analytes other than volatile organic compounds.

Analyte (parent compound, if different), NHANES cycle	Exposure guidance		BE or other biomarker screening value and matrix	NER data	
	Type, data source, yeara	Value (mg/kg-day)		GM	95th percentile
Acrylamide hemoglobin adducts (acrylamide), 2003–2004	RfD, U.S. EPA 2010	2 × 10–3	190 pmol/g hemoglobinb		
Nonsmokers				49.9	89.6
Smokers				109.9	274
Bisphenol A, 2007–2008	RfD, U.S. EPA 1993	0.05	2,000 μg/L urinec	2.08	13
Triclosan, 2007–2008	RfD, U.S. EPA 2008	0.3	6,400 μg/L urined	15.3	494
Pentachlorophenol, 2001–2002	HBM-I, German HBC 1997		25 μg/L urinee	< LOD (0.5)	1.94
Phthalates, 2007–2008					
Mono-ethylphthalate (diethyl phthalate)	RfD, U.S. EPA 1993	0.8	18,000 μg/L urinef	137	1,790
Mono-n-butyl phthalate (dibutyl phthalate)	RfD, U.S. EPA 1990	0.1	2,700 μg/L urinef	18.9	110
Mono-benzylphthalate (benzyl butyl phthalate)	RfD, U.S. EPA 1993	0.2	3,800 μg/L urinef	10	81.4
Sum of 4 metabolites of DEHP	RfD, U.S. EPA 1991	0.02	400 μg/L urineg	96.5	1,019
Mono-carboxyoctylphthalate (di-isononylphthalate)	ADI, CPSC 2001	0.12	390 μg/L urineh	6.8	63
Persistent organohalogen compounds, 2003–2004					
Hexachlorobenzene	MRL, ATSDR 2002	5 × 10–4	47 ng/g serum lipidi	15.2	28.9
DDT + DDE, 2003–2004, by age (years)	RfD, U.S. EPA 1996	5 × 10–4	5,000 ng/g serum lipidj		
12–19				99k	529
20–39				141k	694
40–59				285k	1,742
≥ 60				565k	3,980
Dioxin TEQ (29 dioxin, furan, and coplanar PCB compounds), 2003–2004, by age (years)	RfD, U.S. EPA 2011	7 × 10–10	Variable by age due to accumulation; pg/g serum lipidl	< LOD (variable)	37.8
12–19			15		14
20–39			21		18.7
40–59			21		32
≥ 60			21		63.2
Summed PCBs (35 congeners), 2003–2004, by age (years)	“Critical concentrations,” ANSES 2010	NA	700 (infants, children, women of childbearing age); 1,800 (other adults); ng/g serum lipidm		
12–19			700	54.4	139
20–39			700	79.2	226.5
40–59			1,800	186.4	470.7
≥ 60			1,800	347.3	929.4
PBDE-99	RfD, U.S. EPA 2008	1 × 10–4	520 ng/g serum lipidn	< LOD (variable)	42.2
Metals					
Cadmium, 2003–2004	RfD, U.S. EPA 1994	5 × 10–4	1.5 μg/L urineo		
Nonsmokers				0.2	0.9
Smokers				0.3	1.6
Sum, DMA + MMA (arsenic, inorganic), 2009–2010	RfD, U.S. EPA 1993	3 × 10–4	5.8 μg/L urinep	4.7	18.9
Mercury, 2007–2008	NRC benchmark concentration assessment, NRC 2000	NA	5.8 μg/L bloodq	0.77	4.64
Thallium, 2007–2008	HBM-I, German HBC 2011	NA	5 μg/L urinee	0.146	0.4
Current-use pesticides, 2001–2002					
2,4-Dichlorophenoxyacetic acid	RfD, U.S. EPA 2011	0.05	2,000 μg/L uriner	< LOD (0.2)	1.27
cis-3-(2,2-Dibromovinyl)-2,2-dimethylcyclopropane carboxylic acid (deltamethrin)	RfD, U.S. EPA 2010	0.01 (adults)	50 μg/L urines	< LOD (0.1)	< LOD (0.1)
4-Fluoro-3-phenoxy-benzoic acid (cyfluthrin)	RfD, U.S. EPA 2002	0.024	240 μg/L urinet	< LOD (0.2)	< LOD (0.2)
Abbreviations: ANSES, Agence nationale de securite sanitaire Alimentation Environnement Travail; ATSDR, Agency for Toxic Substances and Disease Registry; CPSC, Consumer Product Safety Commission; DEHP, di-2(ethylhexylphthalate); HBC, Human Biomonitoring Commission; LOD, limit of detection; MRL, minimal risk level; NA, not applicable; PCB, polychlorinated biphenyl; TEQ, toxic equvalency. GMs and 95th percentiles are reported as point estimates; confidence limits on these estimates are available in the NER. For concentrations < LOD, the LOD is given in parentheses. aAll U.S. EPA exposure guidance values from the Integrated Risk Information System (U.S. EPA 2012a) unless otherwise noted. bDerived based on methods described by Hays and Aylward (2008) with updated U.S. EPA RfD value. cKrishnan et al. (2010a). dKrishnan et al. (2010b). eGerman HBC (2012), derived from occupational biomonitoring data—no exposure guidance value was derived. fAylward et al. (2009a). gAylward et al. (2009b). hADI from CPSC (2001); BE derivation by Hays et al. (2011). iMRL from ATSDR (2012), BE derivation presented by Aylward et al. (2010a). jKirman et al. (2011). kMedians. lBased on U.S. EPA (2012c) RfD for dioxin as based on neonatal thyroid hormone alterations. Serum lipid concentrations associated with chronic intake at the RfD were modeled using the U.S. EPA (2012c) approach. Identification of appropriate BE values for children under 12 years of age would require additional modeling and considerations. Age-specific NER concentration data as reported by Patterson et al. (2009). mCritical concentrations from ANSES (2010). Age-specific NER concentration data as reported by Patterson et al. (2009). nKrishnan et al. (2011). oHays et al. (2008b). pHays et al. (2010; DMA+MMA only due to low detection rates for inorganic arsenic species). qNRC 2000—benchmark concentration in blood divided by uncertainty factor of 10. rRfD updated November 2011 by U.S. EPA (2011). BE based on Aylward and Hays (2008), but reflecting updated RfD, which was increased by a factor of 10 due to removal of the 10-fold uncertainty factor related to database uncertainties. sRfD as described by U.S. EPA (2010); BE derivation presented by Aylward et al. (2011). tRfD as described by U.S. EPA (2002); BE derivation presented by Hays et al. (2009).					

Here we present an initial examination of the broad range of chemicals included in the NER, comparing the measured levels in the NER to the risk assessment–based BE values as well as other risk assessment based biomarker values. These comparisons can be used to inform decisions on prioritizing additional research and prioritizing national strategies to reduce exposures. These comparisons can also be used to identify data needs to enable a fuller assessment of the NER biomonitoring data in a health risk context.

## Methods

*NER biomonitoring data.* We obtained descriptive statistics for the NER biomonitoring data from the CDC online summary tables (CDC 2012). The most recent available data were selected for each analyte. For some analytes with previously described dependence of biomarker concentration on age (some persistent organochlorine compounds) or smoking status (e.g., cadmium, acrylamide, benzene, toluene), simple descriptive statistics for population groups [i.e., the population weighted geometric mean (GM) and 95th percentile] were calculated from online data available from the National Health and Nutrition Examination Survey (NHANES) (CDC 2012) using STATA IC10 (StataCorp, College Station, TX). Such population group analyses were conducted where *a priori* information suggested relevance, but no attempt was made at a comprehensive assessment of patterns by smoking or age across all the chemicals in the analysis. For analysis of acrylamide and cadmium biomarkers, a serum cotinine concentration of ≥ 10 ng/mL was assumed to indicate that the individual was a smoker (Pirkle et al. 1996). For volatile organic compounds (VOCs), we used the analysis by smoking status presented in Kirman et al. (2012) based on the presence or absence of detectable serum 2,5-dimethylfuran.

*Biomarker screening values.* Chemical-specific, public health–based screening values for the evaluation of biomarker concentrations were identified from several sources. These included the following: BEs (reviewed by Angerer et al. 2011); Human Biomonitoring-I (HBM-I) values from the German Human Biomonitoring Commission [(2012); reviewed by Schulz et al. (2011)]; a blood concentration RfD equivalent for methylmercury derived from the National Research Council (NRC 2000) report; and “critical concentrations” for polychlorinated biphenyl compounds recently set by the French Agency for Food, Environmental and Occupational Health and Safety [Agence nationale de securite sanitaire Alimentation Environnement Travail (ANSES) 2010]. Where more than one screening value was available, we selected one based on a hierarchy of preference. For example, BE values have been derived for bisphenol A based on multiple exposure guidance values such as an RfD from the U.S. Environmental Protection Agency (EPA) and a TDI set by the European Food Safety Authority. First preference was given to BE values corresponding to the U.S. EPA RfD or reference concentration (RfC) values because they comprise a large number of detailed, peer-reviewed, publicly available assessments (U.S. EPA 2012a); this was followed by the BE values corresponding to Agency for Toxic Substances and Disease Registry (ATSDR) minimal risk levels (MRLs; ATSDR 2012). For VOCs, BE values corresponding to inhalation-based exposure guidance values were selected when available, followed by those corresponding to oral exposure guidance values when no inhalation-based value was available. For several compounds, including dioxins, 2,4-dichlorophenoxyacetic acid, and six VOCs (Table 2), new risk assessments have been published since the BE values were derived and published. For these chemicals, the BE values were updated to correspond to the revised risk assessments, and the new values are footnoted in the results tables.

**Table 2:** Risk assessment exposure guidance values (with year of derivation), corresponding screening BEs, and NHANES data for VOCs from the 2003–2004 cycle.

Chemical	Exposure guidance		BE value (μg/L whole blood)	NER 2003–2004 data (μg/L whole blood)	
	Type, data source, yeara	Value		GM	95th percentile
Benzene	RfC, U.S. EPA 2003	0.03 mg/m3	0.15b		
Smokers				0.136	0.44
Nonsmokers				< LOD (0.024)	0.06
Ethylbenzene	MRL, ATSDR 2010	0.25 mg/m3	1c		
Smokers				0.067	0.16
Nonsmokers				0.028	0.071
Styrene	RfC, U.S. EPA 1993	1 mg/m3	3c		
Smokers				0.068	0.18
Nonsmokers				< LOD (0.03)	0.068
Toluene	RfC, U.S. EPA 2005	5 mg/m3	20d		
Smokers				0.324	0.99
Nonsmokers				0.082	0.34
Xylenes	RfC, U.S. EPA 2003	0.1 mg/m3	0.3c		
Smokers				0.261	0.6
Nonsmokers				0.161	0.4
Carbon tetrachloride	RfC, U.S. EPA 2010	0.1 mg/m3	0.19e	< LOD (0.005)	< LOD (0.005)
Chlorobenzene	RfD, U.S. EPA 1993	0.02 mg/kg-day	0.2c	< LOD (0.011)	< LOD (0.011)
1,2-Dibromo-3-chloropropane	RfC, U.S. EPA 1991	0.0002 mg/m3	0.001c	< LOD (0.1)	< LOD (0.1)
1,2-Dichlorobenzene	RfD, U.S. EPA 1991	0.09 mg/kg-day	0.7c	< LOD (0.1)	< LOD (0.1)
1,4-Dichlorobenzene	RfC, U.S. EPA 1996	0.8 mg/m3	3c	0.194	3.3
1,1-Dichloroethene	RfC, U.S. EPA 2002	0.2 mg/m3	0.3c	< LOD (0.009)	< LOD (0.009)
cis-1,2-Dichloroethene	RfD, U.S. EPA 2010	0.002 mg/kg-day	0.034e	< LOD (0.01)	< LOD (0.01)
trans-1,2-Dichloroethene	RfD, U.S. EPA 2010	0.02 mg/kg-day	0.07c	< LOD (0.01)	< LOD (0.01)
Dichloromethane	RfC, U.S. EPA 2011	0.6 mg/m3	2e	< LOD (0.07)	< LOD (0.07)
1,2-Dichloropropane	RfC, U.S. EPA 1991	0.004 mg/m3	0.01c	< LOD (0.008)	< LOD (0.008)
Hexachloroethane	RfC, U.S. EPA 2011	0.03 mg/m3	0.2e	< LOD (0.011)	< LOD (0.011)
Methyl-tert-butylether (MTBE)	RfC, U.S. EPA 1993	3 mg/m3	20c	0.011	0.17
Nitrobenzene	RfC, U.S. EPA 2009	0.009 mg/m3	0.03c	< LOD (0.3)	< LOD (0.3)
1,1,2,2-Tetrachloroethane	RfD, U.S. EPA 2010	0.02 mg/kg-day	0.2e	< LOD (0.01)	< LOD (0.01)
Tetrachloroethylene	RfD, U.S. EPA 1988	0.01 mg/kg-day	1c	0.0422	0.14
1,1,1-Trichloroethane	RfC, U.S. EPA 2007	5 mg/m3	20c	< LOD (0.048)	< LOD (0.048)
1,1,2-Trichloroethane	RfD, U.S. EPA 1995	0.004 mg/kg-day	0.05c	< LOD (0.01)	< LOD (0.01)
Trichloroethylene	RfC, U.S. EPA 2011	0.002 mg/m3	0.0062e	< LOD (0.012)	< LOD (0.012)
Chloroform	RfD, U.S. EPA 2001	0.01 mg/kg-day	230 pg/mLf	10 pg/mLg	50 pg/mL
Bromodichloromethane	RfD, U.S. EPA 2005	0.02 mg/kg-day	80 pg/mLf	1.4 pg/mLg	9.5 pg/mL
Dibromochloromethane	RfD, U.S. EPA 2005	0.003 mg/kg-day	20 pg/mLf	< LOD (0.6) pg/mLg	7.2 pg/mL
Bromoform	RfD, U.S. EPA 2005	0.03 mg/kg-day	130 pg/mLf	< LOD (1.5) pg/mLg	6.4 pg/mL
BE values corresponding to inhalation expoure guidance values were used where available; when missing, BE values corresponding to oral exposure guidance values were selected. Point estimates for GM and 95th percentiles are presented; confidence intervals on these statistics are available in the NER. aAll U.S. EPA exposure guidance values available at U.S. EPA (2012a); ATSDR MRL available at ATSDR (2012). bHays et al. (2012). cAylward et al. (2010b). dAylward et al. (2008a). eReflects risk assessment value established since publication of Aylward et al. (2010b). Corresponding steady-state blood concentrations estimated using relationships between constant external exposures and blood concentrations from Table 2 of Aylward et al. (2010b). fAylward et al. (2008b). gMedian.					

For this review, a “screening” value is one that allows evaluation of biomonitoring data in the context of chemical risk assessments. BE values and other values used here are not screening values in the medical sense of the term.

The definitions and methods for deriving BE and HBM-I values were reviewed by Angerer et al. (2011). In general, the identified screening values correspond to biomarker concentrations consistent with exposure levels previously deemed to be unlikely to result in adverse effects in the human population, including sensitive subgroups [e.g., see the U.S. EPA definition of RfD in the U.S. EPA Integrated Risk Information System (IRIS) Glossary (U.S. EPA 2012b)].

*Risk assessment approaches.* NER biomonitoring data were evaluated using the BE or other identified screening values in two ways. For BEs based on noncancer end points (e.g., RfDs, MRLs), hazard quotients (HQs) were calculated as follows:


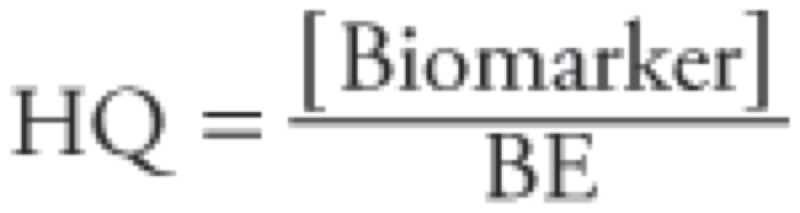
[1]

HQ values near or above 1 provide an indication that exposure levels are near or above the exposure benchmark underlying the BE value.

BE values corresponding to risk-specific doses (BE_RSD_) have been derived as well and can be used in a parallel fashion to evaluate chemicals with slope factors. Risk-specific doses (RSDs) are estimates of the lifetime average daily exposure associated with a specified (cancer) risk level for a chemical. BE_RSD_ values provide an estimate of the lifetime steady-state blood concentration that would result from chronic exposure at those risk-specific doses (the same is true for BEs based on chronic RfDs and MRLs). Risks were estimated assuming linear extrapolation on the basis of biomarker concentrations above and below the BE_RSD_ value. For highly persistent analytes, measured biomarker concentrations at a single point in time may provide a reasonably accurate surrogate for long-term average concentrations and, therefore, potential risks (according to current risk assessments) for individuals. However, for highly transient analytes, conclusions regarding both noncancer and lifetime cancer risks based on samples from a single time point in the blood or urine of individuals are much more uncertain (Aylward et al. 2012).

Some compounds in the NER analyte list were detected in few or no individuals in the sampled population. For those cases in which an analyte was below the limit of detection (LOD) at the GM or 95th percentile in the NER data set, the LOD was compared to the BE values in order to assess whether the LOD is sufficiently sensitive to provide information relevant in a risk assessment context. For instance, for compounds with LOD values < the BE, a lack of detected analytes in the population indicates that exposures in the general population are below the risk assessment–derived exposure guidance values. This information can be useful in assessing whether future biomonitoring studies (with improved detection limits) are likely to be of increased value in a risk assessment context, or whether the LOD is sufficiently sensitive to provide relevant public health risk assessment conclusions for the analyte.

## Results

The descriptive statistics for the NER data and the identified biomarker-based risk assessment values based on noncancer exposure guidance values are summarized in Tables 1 and 2. Chemical-specific biomarker-based screening values were identified for the evaluation of 130 NER analytes [including 29 dioxin, furan, and coplanar polychlorinated biphenyls (PCBs) and 35 non-dioxin-like PCBs]. A number of chemical groups that are included in the NHANES analyte list have few or no screening values available for assessing biomarker concentrations. These include the perfluorinated compounds, the phytoestrogens, most of the polybrominated diphenylethers, most of the pesticide analytes (including the organophosphates), the parabens, and many of the metals.

**Figure 1:**
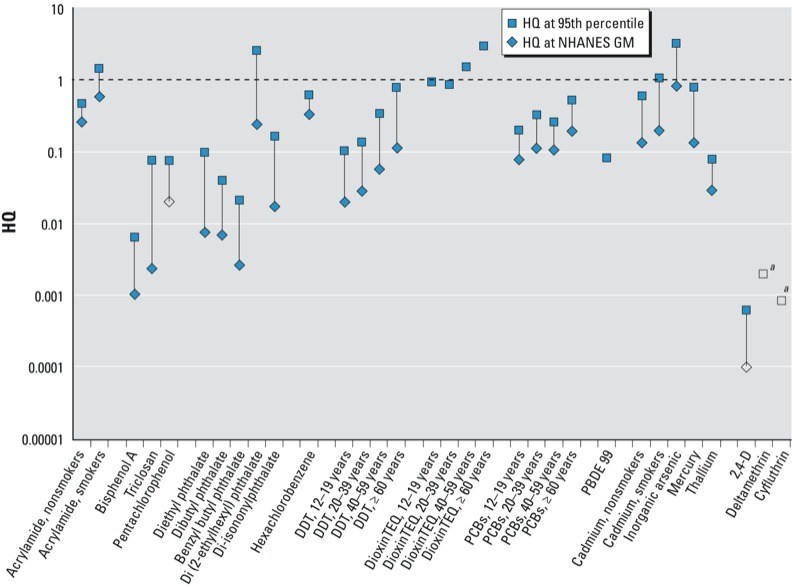
HQs for NER analytes with available BEs or other biomarker-based screening values, excluding VOCs (see Figure 2 for VOCs); screening values and NHANES data reported in Table 1. Open symbols correspond to the HQ at the limit of detection (LOD) in cases where the analyte was not detected in the NHANES survey at the specified quantile. For dioxin toxic equvalency (TEQ) and PBDE-99, concentrations were not quantifiable at the GM, and variable LODs in the NHANES data set prevent selection of a single value to represent LOD. DDT, dioxin TEQ, and PCBs HQs are shown by age in years. ^*a*^Deltamethrin and cyfluthrin were not detected at either the GM or the 95th percentile; the HQ associated with the LOD is indicated in the figure.

**Figure 2:**
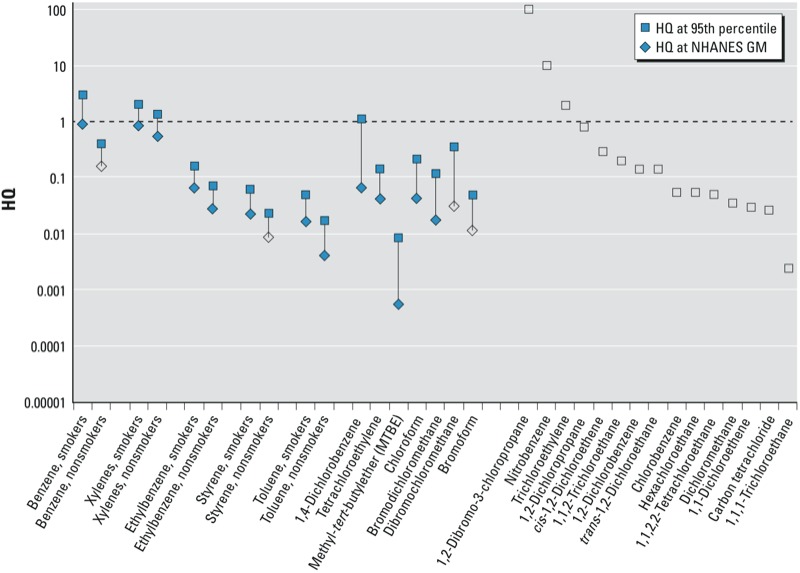
HQs for VOCs from the NHANES 2003–2004 cycle for those VOCs with available BE values (see Table 2). Open symbols correspond to the HQ at the limit of detection in cases where the analyte was not detected in the NHANES survey at the specified quantile.

Calculated HQ values based on noncancer end points are presented in Figure 1 (non-VOCs) and Figure 2 (VOCs). Among the non-VOCs, HQ values approached or exceeded 1 at the population 95th percentiles for acrylamide (in smokers), di(2-ethylhexyl)phthalate (DEHP), dioxins, cadmium (in smokers), and inorganic arsenic. Of these, DEHP and inorganic arsenic have short half-lives in the body and are expected to exhibit substantial intraindividual variability. Thus, upper and lower percentiles of the biomarker concentration distribution for these two chemicals may not be informative of long-term average biomarker concentrations for individuals. That is, spot samples at the upper end of the distribution may represent samples collected closer in time to exposure events rather than necessarily indicating a higher absolute exposure level. Similarly, spot samples with concentrations at the lower end of the distribution may represent samples taken at longer times since exposure events rather than indicating a lower absolute exposure level (Aylward et al. 2012). Central tendency concentrations, however, are still likely to be representative of longer-term average exposure levels for the general population. The levels measured for acrylamide, dioxins, and cadmium are expected to be more stable, with little intraindividual variability. HQ values did not exceed 1 at the GM biomarker concentration for any analyte. HQ values between 0.1 and 1 were observed for a number of these more stable compounds at both the GM and the 95th percentile.

Among VOCs, 95th percentile HQs were > 1 for benzene in smokers, for xylenes in both smokers and nonsmokers, and for 1,4-dichlorobenzene (Figure 2). As noted in previous evaluations of the VOCs (Kirman et al. 2012), these compounds are rapidly metabolized and the measured biomarkers tend to be relatively transient. Upper and lower percentiles of the biomarker concentration distribution may not be informative of daily or longer-term average biomarker concentrations, with the possible exception of smokers. Central tendency measures such as GMs may be more informative of typical average biomarker concentrations in the population. At the GM, no VOC analytes exceeded an HQ of 1.

Many VOC analytes were detected in < 5% of the sampled population (open symbols, right side of Figure 2). For most of these analytes, the LOD was ≤ the BE, suggesting that from a risk assessment perspective the analyses were sufficiently sensitive to provide useful information. In this context, the lack of detectable concentrations suggest that exposures in the general population are typically well below levels associated with risk assessment–based exposure benchmarks. This information can be considered as part of an assessment of the value of dedicating resources (larger biological sample volumes or additional methods development) to attaining lower LODs for these analytes.

**Figure 3:**
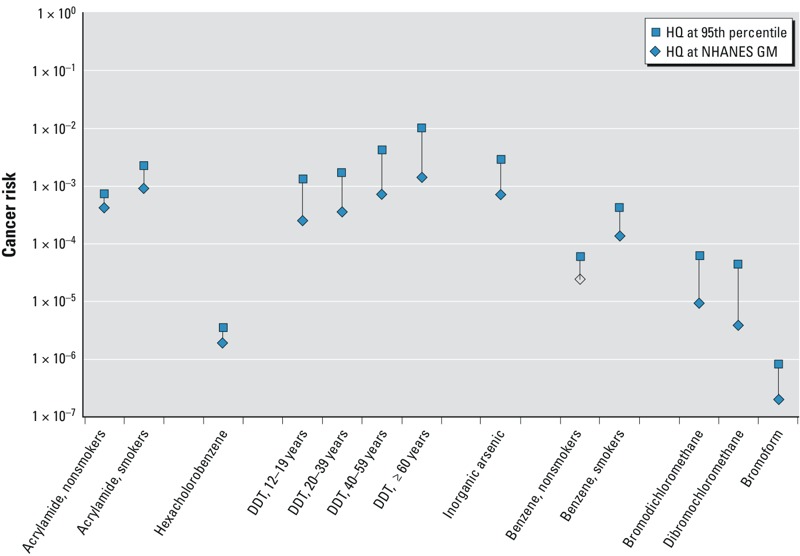
Cancer risk estimates based on biomarker concentrations from the NER data set for those compounds with available cancer-based BE values. Open symbols correspond to the risk level at the limit of detection in cases where the analyte was not detected in the NHANES survey at the specified quantile. All risk estimates assume that biomarker concentrations represent lifetime exposure levels. DDT risk estimates are shown by age in years. [Cancer risk-based BE values are presented by Hays and Aylward 2008 (acrylamide); Aylward et al. 2010a (hexachlorobenzene); Kirman et al. 2011 (DDT); Hays et al. 2010 (arsenic), Hays et al. 2012 (benzene), and Aylward et al. 2008a (trihalomethanes)].

Cancer risk levels corresponding to the GM and 95th percentile NER biomarker concentrations are presented in Figure 3 for the 13 analytes with both cancer risk–based screening values and frequent detections (> 60%) in the NER data sets. Cancer risk level targets in U.S. regulatory arenas generally focus on a range of 10^–6^ to 10^–4^, although this range is flexible depending on the context (e.g., offsetting benefits, widespread vs. infrequent exposures). Cancer risk estimates corresponding to the 95th percentile biomarker concentrations for 12 of the 13 compounds with available cancer-based screening values exceed the 10^–6^ cancer risk level, and cancer risk estimates corresponding to the GM biomarker concentrations for 8 of these compounds approach or exceed the 10^–4^ cancer risk level (see Figure 3).

## Discussion

This review provides the first broad examination of the NER biomonitoring data sets in a health risk context across a broad range of the included analytes. Biomonitoring-based screening values addressing 130 of the NER analytes were identified, allowing the measured concentrations of these biomarkers in the U.S. population to be evaluated in terms of risk assessment–based screening values. HQs > 1 were observed at the 95th percentile for a number of analytes, suggesting exposures in portions of the U.S. population may exceed risk assessment–based exposure guidance values for these compounds, at least intermittently. Evaluation of the health risk implications of HQ values > 1 requires consideration of chemical-specific information on the basis for the underlying exposure guidance values, the uncertainty factors applied in the derivation of those values, the robustness of the health effects database, and other factors. Some of the chemicals with higher HQ values are compounds that are intentionally manufactured and used in products or released to the environment (e.g., 1,4-dichlorobenzene and DEHP). However, others are present in the environment primarily due to formation as unintentional by-products of combustion or other reactions (e.g., dioxin-like chemicals and acrylamide) or at least in part due to their natural occurrence (e.g., arsenic and mercury compounds).

Similarly, biomarker concentrations approached or exceeded concentrations consistent with cancer risk levels > 1 × 10^–6^ for a number of analytes. However, interpretation of risks for cancer should be made cautiously because the risk-specific BE values presented here are estimates of the steady-state concentration associated with lifetime average daily doses at the risk-specific dose.

Comparison of measured biomarker concentrations to BE values incorporates an implicit assumption that the biomarker concentrations represent chronic average biomarker concentrations for the individual persons sampled. While this may be a reasonable assumption for highly persistent compounds, spot sample concentrations of more transient compounds may not provide reliable surrogates for long-term or lifetime average biomarker concentration in individuals (Aylward et al. 2012). For such chemicals, extremes at both ends of the population distribution of biomarker concentrations may be more unreliable as indicators of long-term exposure levels for individuals, while central tendency measures such as GM calculations may be more informative of longer-term average biomarker concentrations on a population basis. The results presented here indicate that, even at the GM biomarker concentrations, cancer risk levels are > 1 × 10^–6^ for several analytes, and, for some analytes, > 1 × 10^–3^.

The chemicals currently on the NER analyte list have been selected for inclusion in the survey for a variety of reasons. Many of the chemicals have traditionally been of concern because of known toxic potency (e.g., the dioxin-like chemicals), high industrial volume (e.g., selected solvents such as toluene), potential for bioaccumulation (e.g., persistent organochlorine insecticides), potential for widespread exposure (e.g., phthalates, trihalomethanes), or combinations of these reasons. Given this combination of selection criteria, the presence of some chemicals near or above the risk assessment–based screening criteria is not unexpected. And, while biomonitoring data for a number of chemicals suggest exposure levels approaching or exceeding the risk assessment–based benchmarks for some of the population, it is also important to note that for most of the chemicals evaluated here the HQ values are < 1 for all or the majority of the population.

*Combined exposures.* A major issue of interest when the levels of multiple analytes are examined is the importance of co-occurrence and the potential for combined exposure and toxicity. While HQ values for individual analytes might not exceed 1 in an individual or in the population, thereby suggesting that adverse effects are unlikely, the presence of multiple chemicals in individual persons raises the question of whether interactions (additive, synergistic, or antagonistic) occur that could result in adverse effects even when HQ values for individual analytes are < 1. Considerations relevant to assessment of combined exposures to chemicals are discussed in the recent International Programme on Chemical Safety (IPCS) *Framework for Assessment of Combined Exposures* (Meek et al. 2011). Factors to be evaluated when considering a combined exposure and risk assessment include whether the chemicals have a common mechanism or mode of action, common toxicity targets, are expected to co-occur, and other considerations.

**Figure 4:**
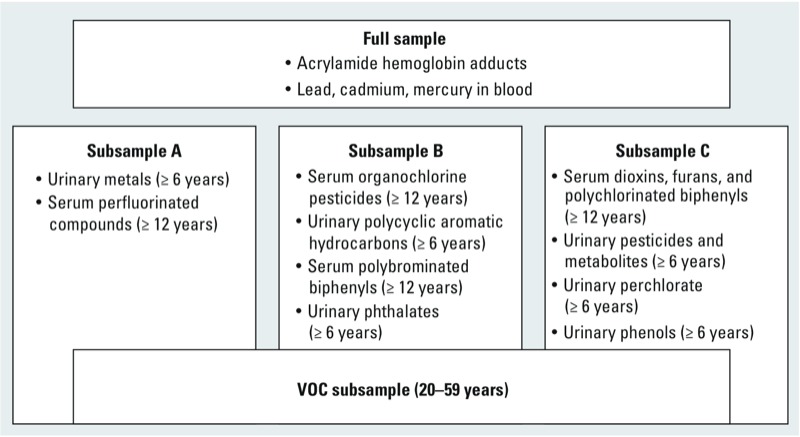
Analytes by NHANES subsample from 2003–2004 cycle. Subsamples A, B, and C represent approximately one-third samples of the full NHANES sample for a given cycle; the VOC subsample overlaps groups A, B, and C. Analytes were measured in blood or urine specimens from persons within the specified subsample and meeting the specified age cutoffs.

The NER data sets provide a nearly unprecedented opportunity to examine co-occurrence of chemicals in biological matrices, reflecting concurrent exposures in the population. However, the full set of analytes in the NER program is not measured in any individual. Instead, because of limitations in the volume of biological samples (i.e., blood, urine) available, analyses for specific analyte groups are generally conducted on one-third subsets of the full NER sample in a given cycle. This still results in multiple chemical groups being measured in many individuals, so it is possible to examine coexposure to selected sets of chemicals in individuals in the data set. Figure 4 presents a schematic showing the distribution of analytes among the NHANES subsets from the 2003–2004 cycle.

Combined exposure assessment may be of particular interest for certain groups of chemicals if the chemicals produce similar pathology or if they act on a similar mechanistic pathway or toxicological end point. For example, the current U.S. EPA noncancer assessments for the chlorinated and brominated trihalomethanes (THMs) are based on similar liver pathology as the most sensitive end point (U.S. EPA 2012a; reviewed by Aylward et al. 2008b). THMs are believed to produce liver toxicity through a similar mode of action, and combined exposure to these chemicals is likely to occur. Under these conditions, it may be appropriate to assess the combined exposures using a hazard index (HI) approach, which assumes dose addition. Thus, for each individual in the NHANES data set, a THM HI was also calculated, summing the chemical-specific HQs across the four THM compounds (*i* = 1 to 4):


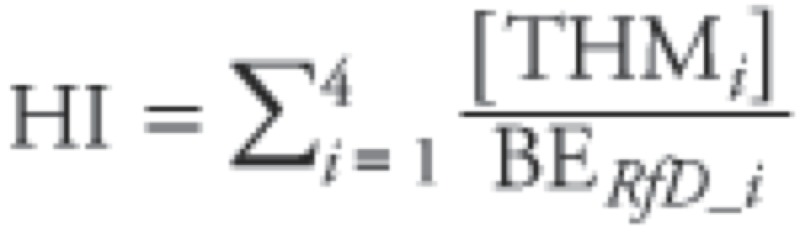
[2]

**Figure 5:**
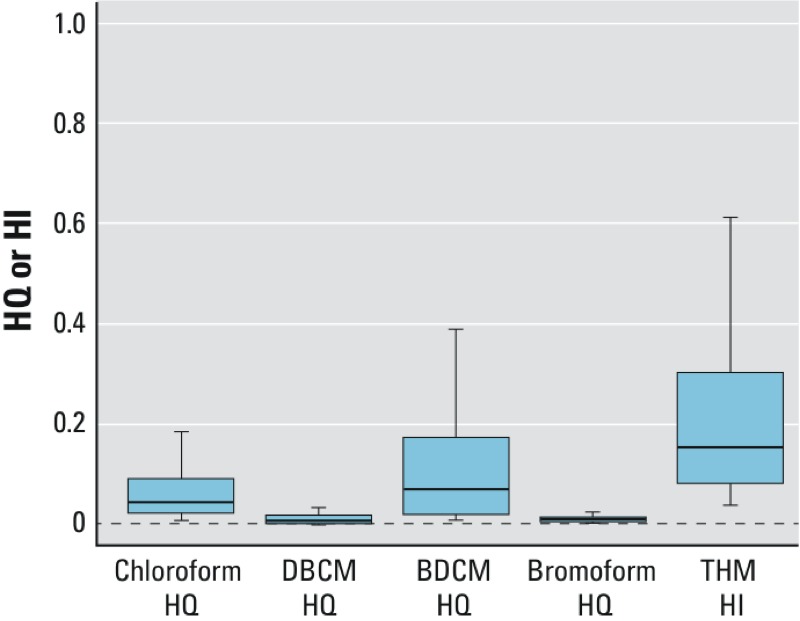
Box plots of HQs for individual THM compounds and the HI for the combined THM HQs calculated per Equation 2. Extreme values are omitted. The horizontal line indicates the median, boxes represent the interquartile range, and lower and upper whiskers extend to 1.5 times the interquartile range below the 25th and above the 75th percentiles, respectively.

For THM compounds in the 2003–2004 NER cycle, HIs for combined exposure to THM compounds (calculated on an individual-by-individual basis) did not exceed 1 at the 95th percentile (Figure 5; see also LaKind et al. 2010).

*Data gaps and limitations.* As discussed above, a significant number of the chemical groups in the NER analyte list have few or no available BEs or other screening values, limiting the overall assessment of the full data set from a risk assessment perspective. Development of additional screening values covering a greater proportion of the NER analyte list, either through direct epidemiological studies linking alterations in health end points to biomarker concentrations, or through derivation of additional biomarker concentrations corresponding to toxicity-based exposure guidance values (as in the Biomonitoring Equivalents framework) would be useful in broadening the perspectives and utility of the evaluation methods presented here. This may be challenging because of several factors: *a*) limited data on pharmacokinetics allowing translation of external to internal exposure levels; *b*) the inclusion of nonspecific biomarkers, including degradates or metabolites that may appear in biological matrices due to direct exposure in the environment; and *c*) a lack of existing risk assessments or method for interpreting hazards of exposure (e.g., phytoestrogens). For some chemicals, there are additional limitations in the ability to assess the NER data because of the limited analytical sensitivity relative to population exposures (e.g., for metabolites of inorganic arsenic).

Additional uncertainties include chemical-specific issues. For example, dioxin concentrations in serum are routinely expressed in terms of dioxin “toxicity equivalents”; however, the relative potency estimates used for these calculations are specifically designed to estimate toxicity of mixtures on an intake basis, not on a tissue or body concentration basis, and differences in congener pharmacokinetics may result in inaccuracies in the serum-based toxic equvalency (TEQ) estimates (Van den Berg et al. 2006; U.S. EPA 2012c). For bioaccumulative compounds, in general, lifetime average daily exposure at a given RSD or RfD would be expected to result in an age-dependent accumulation of the biomarker, and thus consideration of age and accumulation is important.

Finally, as discussed above, the BE values are estimates of biomarker concentrations consistent with specific existing risk assessment–derived exposure guidance values such as RfDs and MRLs; reliance on exposure guidance values other than those selected in this analysis could result in different BE values and estimated HQ or cancer risk estimates. Such exposure guidance values are the result of a risk assessment process that often involves extrapolation of toxicity data from laboratory animals to humans and the application of uncertainty factors to account for possible differences between animals and humans and among individuals in the human population, and these values are exposure route specific. The BE values rely upon available toxicokinetic data to estimate corresponding steady-state biomarker concentrations, with attendant uncertainties. Biomonitoring data often reflect multiple exposure routes and pathways that may or may not correspond to the exposure routes assumed in the underlying risk assessment, and the data reflect concentrations at a point in time that may be more or less representative of long term average concentrations, depending upon the chemical and exposure pathways. These uncertainties and complexities are important considerations in the examination and interpretation of the results presented in this analysis and should be incorporated in more detailed examination of the biomarker data and assessment of potential health risks on a chemical-specific basis.

## Conclusions

The exposure data provided by the NER biomonitoring program are unique in terms of providing a cross-chemical assessment of the U.S. population’s exposures to chemicals. Many approaches to evaluating and using these data for public health research are possible. This approach, in which these data are assessed in comparison to the available BE values and related health risk–based screening values, provides for the first time a means for examining population exposures to multiple environmental chemicals in the context of the risk assessments for those chemicals. This evaluation allows, for the chemicals included, a comparative analysis that can assist risk managers in prioritization of chemicals for more detailed chemical-specific evaluation and risk assessment follow-up. Such activities may include exposure pathway studies, detailed evaluation of underlying toxicological or risk assessment data and uncertainty factors included in the risk assessment process, and active steps to identify exposure mitigation strategies where appropriate. The value of the data will increase as BE values or other health risk–based screening values are developed for additional analytes, which will allow expansion of the subset of NER analytes that can be placed into this context.
